# Target-driven machine learning-enabled virtual screening (TAME-VS) platform for early-stage hit identification

**DOI:** 10.3389/fmolb.2023.1163536

**Published:** 2023-03-13

**Authors:** Yuemin Bian, Jason J. Kwon, Cong Liu, Enrico Margiotta, Mrinal Shekhar, Alexandra E. Gould

**Affiliations:** ^1^ Center for the Development of Therapeutics, Broad Institute of MIT and Harvard, Cambridge, MA, United States; ^2^ Cancer Program, Broad Institute of MIT and Harvard, Cambridge, MA, United States; ^3^ Department of Medical Oncology, Dana-Farber Cancer Institute, Boston, MA, United States; ^4^ Harvard Medical School, Boston, MA, United States

**Keywords:** hit identification, virtual screening, machine learning, drug discovery, AIDD

## Abstract

High-throughput screening (HTS) methods enable the empirical evaluation of a large scale of compounds and can be augmented by virtual screening (VS) techniques to save time and money by using potential active compounds for experimental testing. Structure-based and ligand-based virtual screening approaches have been extensively studied and applied in drug discovery practice with proven outcomes in advancing candidate molecules. However, the experimental data required for VS are expensive, and hit identification in an effective and efficient manner is particularly challenging during early-stage drug discovery for novel protein targets. Herein, we present our TArget-driven Machine learning-Enabled VS (TAME-VS) platform, which leverages existing chemical databases of bioactive molecules to modularly facilitate hit finding. Our methodology enables bespoke hit identification campaigns through a user-defined protein target. The input target ID is used to perform a homology-based target expansion, followed by compound retrieval from a large compilation of molecules with experimentally validated activity. Compounds are subsequently vectorized and adopted for machine learning (ML) model training. These machine learning models are deployed to perform model-based inferential virtual screening, and compounds are nominated based on predicted activity. Our platform was retrospectively validated across ten diverse protein targets and demonstrated clear predictive power. The implemented methodology provides a flexible and efficient approach that is accessible to a wide range of users. The TAME-VS platform is publicly available at https://github.com/bymgood/Target-driven-ML-enabled-VS to facilitate early-stage hit identification.

## 1 Introduction

Drug discovery is expensive. Considering a representative target portfolio, high-throughput screening (HTS) is presently the most widely applicable technology for delivering chemical entry points for drug discovery campaigns (Scannell et al., 2022), but despite its popularity, this high-cost method can result in low hit rates (Zeng et al., 2020). The attrition rates of identified hits are further increased during the validation phase and optimization stage due to inherent deficits in the absorption, distribution, metabolism, excretion, and toxicity (ADMET) properties (Feinberg et al., 2020; Xiong et al., 2021). Such challenges emphasize the demand for additional approaches that can, in parallel, perform a low-cost and efficient screening to identify potential hits and discard inappropriate structures. Thus, the strategy of exploiting the computational power of *in silico* virtual screening (VS) was proposed as a coherent solution.

In VS efforts, structure-based and ligand-based approaches serve as two commonly used strategies. Structural data of proteins can aid in computational approaches to infer receptor–ligand interactions within target binding pockets and enable structure-based virtual screening (SBVS) (Shimada et al., 2019; Alon et al., 2021; Jumper et al., 2021; Akdel et al., 2022). SBVS can screen millions of molecules from large-scale compound libraries against protein structures (Lyu et al., 2019; Wang et al., 2019; Graff et al., 2021) and can be further augmented by integrating machine learning (ML) methods that unlock the capacity to screen an ultra-large chemical space (>1 billion compounds) (Lyu et al., 2019; Gentile et al., 2020; Gorgulla et al., 2020; Graff et al., 2021). Ligand-based virtual screening (LBVS) is another commonly used VS strategy where the chemical structures of known active compounds are used to generate a structure–activity model, which is then exploited to identify other molecules that potentially share similar bioactivity. The generation of large-scale chemical databases of bioactive molecules, like ChEMBL (Mendez et al., 2019), serves as a resource to further enable LBVS. Like SBVS, the integration of ML to boost LBVS capabilities has recently grown in popularity with rapid advancements in ML methods and the ever-increasing wealth of large datasets that have been generated (Jing et al., 2018; Vamathevan et al., 2019; Yang et al., 2019; Bian and Xie, 2021; Jiang et al., 2021). ML-integrated LBVS can provide a better understanding of chemical space through latent representations of the chemical properties to predict novel compound activity (Bian et al., 2019a; Bian et al., 2019b; Stokes et al., 2020; Bian and Xie, 2022).

However, the generation of prerequisite datasets to enable VS is non-trivial. SBVS requires the structural information of the target, while protein preparation and crystallography are not facile tasks. LBVS necessitates known ligands with bioactivity data, which often do not exist. To address this demanding situation, we present the TArget-driven Machine learning-Enabled VS (TAME-VS) platform. The platform simply requires the input of a protein target ID and utilizes seven automated, customizable modules to assess compound libraries to identify potential hits. The platform expands the focus of the VS from the target of interest to a broader collection of proteins that share target functions or certain sequence homology. Augmented cheminformatics data are assessed against the expanded protein collection. Supervised machine learning classifiers are subsequently trained after labeling the fetched data and are used to screen future compounds. Herein, we provide further details on method implementation and discuss the results from retrospective case studies across a diverse set of protein targets. Our platform is built to be flexible, simple to use, and enable rapid evaluation of compound databases in a comprehensive manner. This methodology offers an opportunity to augment drug discovery efforts and can increase the accessibility of VS methods for both big and small organizations, and for both computational and experimental scientists.

## 2 Methods and platform implementation

### 2.1 Overall workflow

**FIGURE 1:**
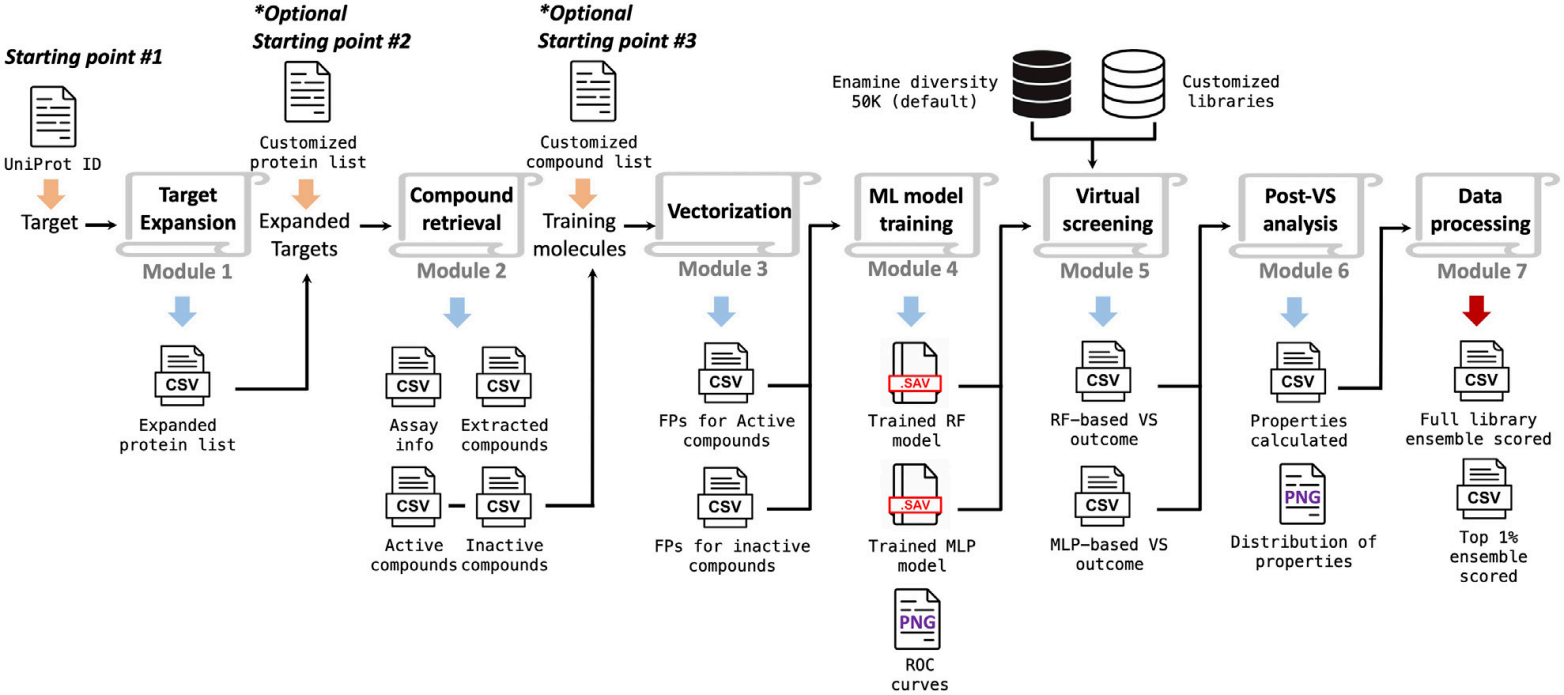
Scheme of the workflow implemented in the target-driven, ML-enabled VS platform.

The overall implementation of the TAME-VS is illustrated in Figure 1. There are three alternative starting points and seven modules in sequence. With only the UniProt ID of the target of interest as the input, the workflow can be initiated from *Starting Point #1*. The first module, *Target Expansion*, performs a global protein sequence homology search through the Basic Local Alignment Search Tool (BLAST) (Altschul et al., 1990) and expands the target list by identifying proteins with high sequence similarities within categorical protein family members. The second module, *Compound Retrieval*, extracts corresponding compounds with activity against the proteins in the target list by querying the ChEMBL database. The extracted compounds are grouped into active and inactive ligands according to assay types and activity cutoffs. The descriptive features of extracted compounds are subsequently converted to chemical fingerprints in the third module, *Vectorization*. The fourth module, *ML Model Training*, trains supervised ML classification models, by default, random forest (RF) and multilayer perceptron (MLP), based on the calculated fingerprints. In the fifth module, *Virtual Screening*, the trained machine learning models are applied to screen user-defined compound collections. By default, an Enamine diversity 50K library is screened, and compounds are ranked according to the prediction scores. Module 6, *Post-VS Analysis*, evaluates quantitative drug-likeness (QED) and calculates key physical–chemical properties for screened compounds. Finally, module 7, *Data Processing*, encapsulates all the data and presents the virtual hits in addition to the evaluation outcome of the entire chemical library in a summary report. Users can also initiate the workflow with their own customized target lists (pre-selected positive targets based on biological rationale) or compound lists (pre-selected active and inactive compounds) from *Starting Point #2* and *Starting Point #3*, respectively. In addition, each module can be used individually, and the output of each module is exported to the corresponding folders for users to review.

The platform is comprehensive yet flexible. It is designed to provide an ML-enabled solution for handling early-stage hit identification. The open-source package of the TAME-VS platform is publicly available on GitHub, together with instructions on setting up the system (https://github.com/bymgood/Target-driven-ML-enabled-VS). The details for each module are discussed in the following paragraphs.

### 2.2 Module 1: Target Expansion

The purpose of *Target Expansion* is to broaden the cheminformatics investigation from the single-query target protein to a broader group of sequence-similar target proteins, based on the hypothesis that proteins with high sequence similarity may possess related structural features and may have an increased likelihood of sharing active ligands. A protein BLAST (BLASTp suite) global search is used to identify proteins with high sequence similarity to the query target through the Biopython package (Cock et al., 2009). The function *NCBIWWW* is imported from *Bio.Blast*. The default sequence similarity cutoff is set at 40% but can be user-defined for a custom similarity cutoff. The arguments *program* and *entrez_query* are set to *BLASTp* and *txid9606[ORGN]* (*homo sapiens*). A table of expanded proteins, including collected target gene names, UniProt IDs, and percent identities are shown in the folder.

### 2.3 Module 2: Compound Retrieval

The purpose of *Compound Retrieval* is to extract reported active and inactive ligands for the expanded target list from publicly available cheminformatics databases, such as ChEMBL, which documents 2.3 M compounds across 13 K targets. ChEMBL is utilized in this module and is accessed using the Python package chembl_webresource_client. The largest experimental datatype for a given protein is utilized to distinguish active and inactive compounds. The default activity cutoff is 1,000 nM for biochemical or biophysical activity (*K*_*i*_, *IC*_*50*_, and *EC*_*50*_), and the default activity cutoff for percentage inhibition (*%INH*) is 50%, with the option for users to define specific cutoff values. It is recommended to check if the *%INH* came from consistent compound concentrations. The folder contains a table summarizing the extracted compounds and their experimental value types, in addition to tables with standard activity values, standard activity value units, SMILES strings, InChI keys, and the associated protein UniProt ID for active and inactive compounds.

### 2.4 Module 3: Vectorization

*Vectorization* is deployed to compute the selected types of molecular fingerprints for the extracted compounds. Different types of fingerprints evaluate the properties of the compounds from various aspects. The platform is designed to enable users to explore various types of fingerprints to evaluate the performance of the trained models using the Cheminformatics package RDKit (Landrum, 2006). Four types of fingerprints -Morgan, AtomPair, Topological and Torsion, and MACCS - are available to choose from in this module. Morgan fingerprints enumerate all circular fragments from each selected center-heavy atom up to the given radius of two atoms. The calculation is realized through *get_morganfp*. AtomPair fingerprints encode each atom as a type to enumerate all distances between pairs. The calculation is realized through *get_AtomPairfp*. Topological and Torsion fingerprints describe a linear sequence of four consecutively bonded non-hydrogen atoms, each described by its atomic type, the number of non-hydrogen branches attached to it, and its number of x electron pairs. Topological and torsion fingerprints are calculated with *get_TopologicalTorsionfp.* The MACCS fingerprint consists of 166 MDL substructure keys, which are calculated from the molecular graph. The calculation is realized through *get_MACCS*. The number of bits, which is an adjustable parameter, is set to 1,024 by default to hash the string into a fixed-length bit-vector for Morgan, AtomPair, and Topological and Torsion fingerprints. The folder contains tables of calculated fingerprints in bit-vector form for active and inactive compounds.

### 2.5 Module 4: ML Model Training

The *ML Model Training* module is utilized to build the RF and MLP models using the calculated fingerprints from module 3 as input features. These two methods were selected to represent both classic ML algorithms and neural networks, and additional add-on features may be appended in future updates. The Python package scikit-learn (Pedregosa et al., 2011) is employed for RF and MLP model implementation. The function of *RandomUnderSampler* from the package *imblearn.under_sampling* is adopted to perform undersampling to counter potential imbalanced training data of active and inactive compounds (Lemaître et al., 2017). The function *GridSearchCV* from *sklearn.model_selection* is used to determine a preferred set of hyperparameters for trained models. The hyperparameter grid for RF includes *n_estimators* (50, 100, and 200) and *max_depth* (4, 6, 10, and 12). The hyperparameter grid for MLP includes *hidden_layer_sizes* [(50, 50, 50), (50, 50), and (50)]; *activation* (tanh and relu); and *alpha* (0.01 and 0.0001). As a concise evaluation for the trained models, ten-fold cross-validation is integrated, and figures of the receiver operating characteristic curve (ROC) for each model are exported for visual inspection. By default, both RF and MLP models are trained, but the user may select a specific model to be prepared. The trained prediction models are shown in the folder.

### 2.6 Module 5: Virtual Screening

The purpose of *Virtual Screening* is to screen the user-defined compound collection using trained machine-learning models. By default, the Enamine diversity 50K library will be screened. An extra Python script, *Library_preparation.py*, is also attached in module 5 for preparing any user-defined libraries into a standard format for this platform. Trained models are loaded and screened in sequence. Compound prediction scores are written out separately for each model.

### 2.7 Module 6: Post-VS Analysis

The *Post-VS Analysis* module evaluates and compares the screening library, with an emphasis on the top 1% of virtual hits to the training set from the perspective of drug-likeness and physical–chemical properties. Distributions of prediction scores from both RF and MLP models are plotted. Quantitative estimate of drug-likeness (QED) (Bickerton et al., 2012), molecular weight (M.W.), LogP, number of H-bond acceptors, number of H-bond donors, and number of rotatable bonds are calculated using functions *Descriptors.TPSA*, *Descriptors.MolWt*, *Descriptors.MolLogP*, *Descriptors.NumHAccepto*, *Descriptors.NumHDonors*, and *Descriptors.NumRotatableBonds* in RDKit, respectively. Data tables including these calculated properties are exported, and distribution plots are prepared to facilitate an intuitional visual inspection.

### 2.8 Module 7: Data Processing

In the final module, *Data Processing*, the selected compounds from the previous modules are consolidated and summarized, and a final list of suggested top virtual hits is reported. An ensemble ranking of molecules is calculated by averaging two individual rankings by RF and MLP. The top 1% of compounds from RF and MLP models and the ensemble ranking are merged. Duplicates are removed as some molecules can be selected as top-ranked by more than one algorithm. Both the full compound list and the top 1% virtual hit list are shown in the folder.

## 3 Results

### 3.1 A case study of applying the platform to stromelysin-2

**FIGURE 2:**
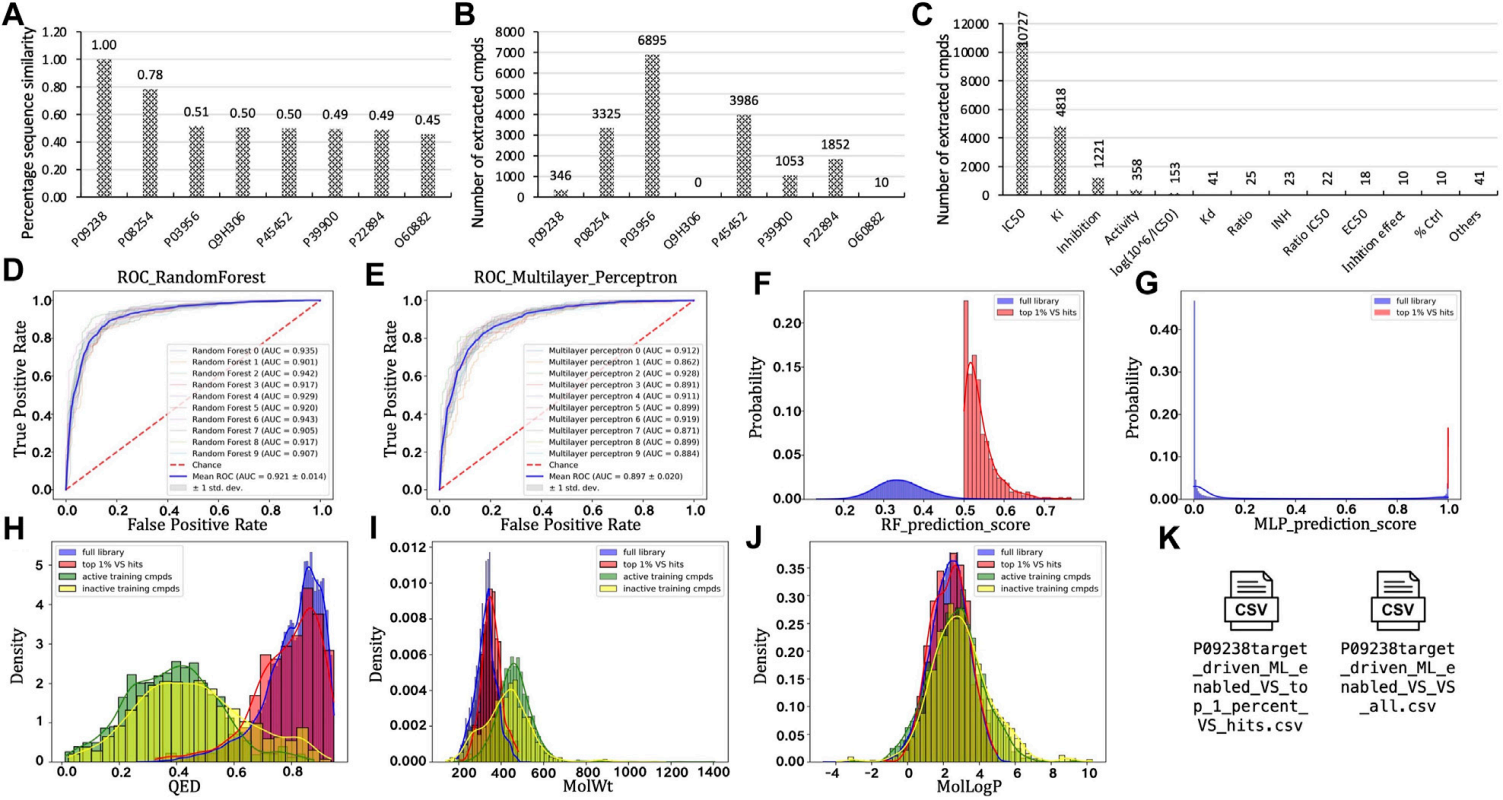
Applying target-driven, ML-enabled VS toward stromelysin-2 (UniProt ID: P09238) as a case study to exemplify outcomes from each module. **(A)**. Protein list after target expansion. **(B)**. Number of extracted compounds for each target in the protein list. **(C)**. Distribution of experimental value types. **(D)**. ROC curve for RF model training. **(E)**. ROC curve for MLP model training. **(F)**. Distribution of prediction scores on the Enamine Diversity 50K library using the RF model. **(G)**. Distribution of prediction scores on the Enamine Diversity 50K library using the MLP model. **(H)**. Distribution of calculated QED. **(I)**. Distribution of calculated MW. **(J)**. Distribution of calculated LogP. **(K)**. Exemplified final reports.

As an exemplified use case of the TAME-VS platform, we chose stromelysin-2 to illustrate the performance and output results of the modules (Figure 2). Stromelysin-2, also known as MMP10, is a proteolytic enzyme belonging to the matrix metalloproteinase (MMP) family that is known to break down extracellular matrix proteins and is involved in tissue remodeling, angiogenesis, and inflammation (Vaalamo et al., 1998; Saghizadeh et al., 2001; Krampert et al., 2004; Koller et al., 2012; Rohani et al., 2015). We entered the UniProt ID, P09238, as an input in *Starting Point #1*. Seven targets that share sequence similarity of over 40% were identified and written down (Figure 2A). A total of 17,467 chemical records were retrieved with activity across all collected targets (Figure 2B). The retrieved chemical records were distributed among a variety of experimental data types, including biochemical and competitive binding. The biochemical assay data type *IC*_*50*_, which contains the most records (10,727 records; see Figure 2C), was utilized to split curated compounds into active and inactive ones at a default concentration of 1000 nM. After the vectorization, the RF (Figure 2D) and MLP (Figure 2E) classification models were trained to distinguish active molecules from inactive ones. ROC curves of ten-time cross-validation provided an intuitional visualization of the robustness of the training process. The Enamine Diversity 50K library was then screened and scored separately by the trained RF (Figure 2F) and MLP (Figure 2G) models. After the virtual screening, properties like QED (Figure 2H), MW (Figure 2I), and LogP (Figure 2J) were calculated and plotted. Eventually, the scored full compound list and top 1% virtual hit list were written on the disk. The overall process took approximately 30 min to finish on a MacBook Pro equipped with a 2.6 GHz 6-Core Intel Core i7 processor.

### 3.2 Retrospective studies on diverse protein targets

In addition to evaluating the efficiency of our platform, we sought to address the effectiveness and performance of our pipeline across a range of protein types. Ten targets representing divergent protein categories, including GPCRs, ligases, oxidoreductases, proteases, kinases, phosphatases, and voltage-gated ion channels, were selected for these studies (Table 1). Using *Starting Point #1*, we performed retrospective VS studies on ten diverse protein targets using their UniProt IDs as the input and assessed if the platform could determine *ex post facto* known active compounds of targets over the broad range of chemical matter represented in the Enamine diversity 50K library. After target expansion and compound retrieval, we observed a wide range of identified homologous targets, retrieved known chemicals, and miscellaneous experimental assay types.

**TABLE 1:** Ten protein targets in retrospective validations.

Target	UniProt ID	Category	# of homologous targets identified	# of reported molecules retrieved	Assay type that gives most records
A2b	P29275	GPCR	2	36629	*K*_*i*_
ACC1	Q13085	Ligase	1	4268	*IC*_*50*_
AKR1B10	O60218	Oxidoreductase	9	4449	*IC*_*50*_
CTSG	P08311	Protease	3	3076	*IC*_*50*_
JAK3	P52333	Kinase	3	32951	*IC*_*50*_
MMP10	P09238	Protease	7	17467	*IC*_*50*_
PRKD1	Q15139	Kinase	2	6981	Inhibition
PTN6	P29350	Phosphatase	1	2434	*IC*_*50*_
RPS6KA3	P51812	Kinase	5	16383	Inhibition
SCN4A	P35499	Voltage-gated ion channel	9	13594	*IC*_*50*_

**FIGURE 3:**
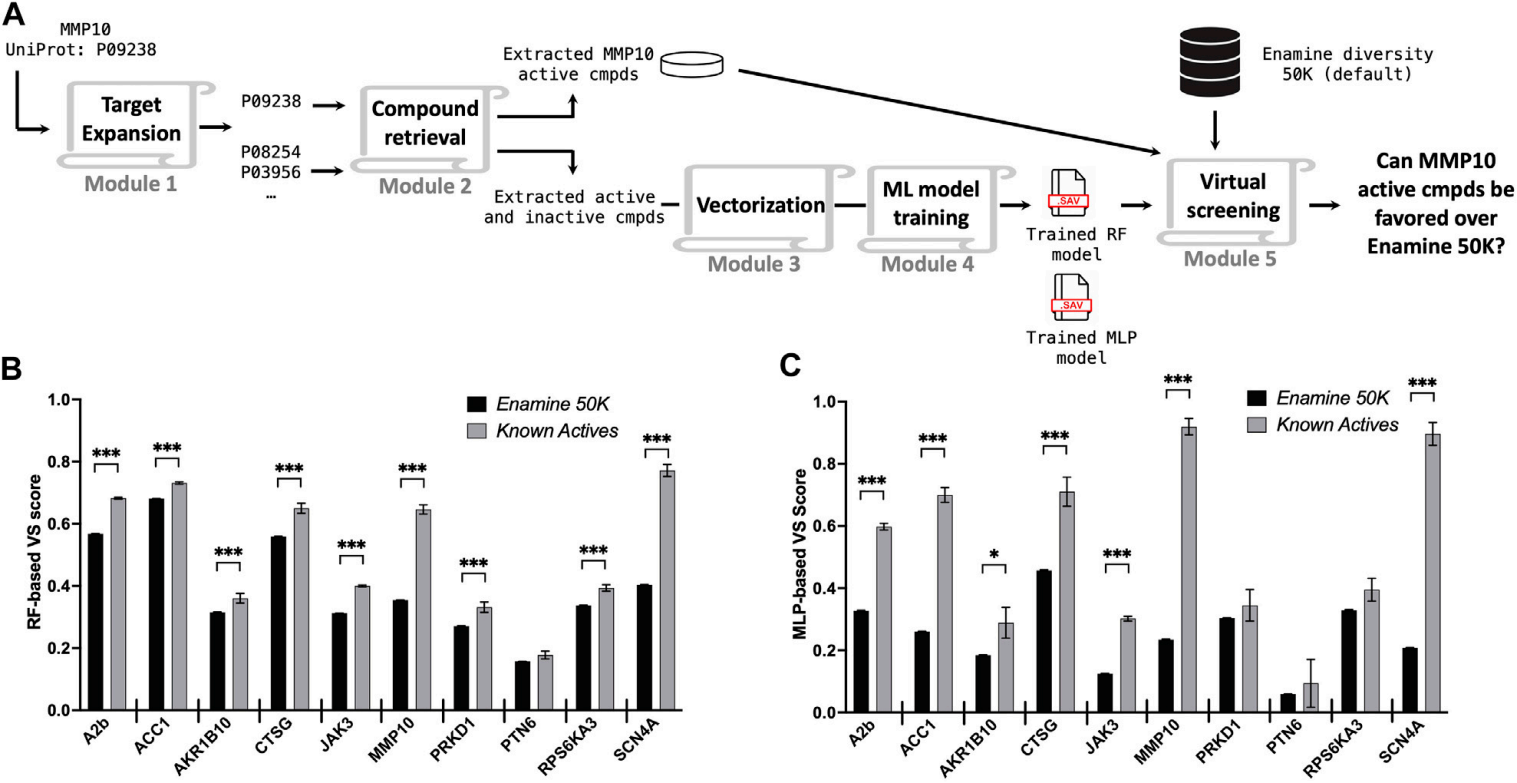
Retrospective validations across ten different protein targets. **(A)** Schematic illustration of a retrospective study. Averaged VS scores reported by the RF model **(B)** and the MLP model **(C)** for the entire Enamine diversity 50K library (black bar) and known active compounds (gray bar) of the query target. The error bar represents the standard error of the mean (SEM), *p < 0.05; **p < 0.01; ***p < 0.001.

A hit identification campaign for a novel target typically lacks reported active compounds or probes. To simulate this scenario, known hits of the query protein were withheld during the model training stage but reintroduced for scoring once the models had been trained with chemical data from the expanded protein target list (Figure 3A). The Enamine Diversity 50K library and known active compounds of the query target were evaluated by the trained predictive models, and the outcomes were assessed. Given that compounds in the Enamine diversity 50K library sparsely represent a general drug-like chemical space, the majority of these molecules are anticipated to be assigned relatively low VS scores by the predictive models compared to active compounds. Indeed, we observe that both RF and MLP assign higher VS scores to known active compound sets as compared to the Enamine 50K chemicals (Figures 3B, C), with a high degree of agreement between the two models (Supplementary Figure S1). Specifically, we observe a significant difference in 9/10 targets for RF and 7/10 targets for MLP. MLP was unable to detect a significant difference in known active compounds compared to Enamine 50K compounds for PRKD1 and RPS6KA3 due to the assay type of inhibition (Table 1) utilized for model training, which can suffer from inconsistent compound testing concentrations. MLP had a 3-fold greater variability in the scoring of known active compounds than RF. However, MLP, on average, provided a two-fold greater differential VS score between the Enamine 50K and known active compounds across targets compared to RF (Supplementary Figure S2). As anticipated, the ability of models to discern a difference between the Enamine 50K and known active compounds was correlated with the number of targets identified during the target expansion phase in addition to the number of molecules identified in the compound retrieval phase (Supplementary Figure S1).

To enable the evaluation of model training and performance, six metrics, namely, AUC, precision, recall, specificity, F1 score, geometric mean, and index of balanced accuracy (IBA), were embedded in the TAME-VS platform for evaluating the performance from various aspects. The calculation of these metrics is detailed in the supplementary information. To reflect the imbalanced training data that active compounds are usually minorities, the training process adopted the down-sampling of inactive compounds with 10-time cross-validation. ROC curves with calculated AUC values for cross-validation were plotted automatically after model training for both RF (Supplementary Figure S3) and MLP (Supplementary Figure S4) models. The values of reported precision, recall, specificity, F1 score, geometric mean, and index of balanced accuracy are summarized into tables for both RF (Supplementary Table S1) and MLP (Supplementary Table S2). Across the ten diversified protein targets, calculated metrics suggested that trained RF and MLP models gave robust and equivalent performances on classifications (Supplementary Figure S5). We observed greater variability in AUC as measured by standard deviation and model precision, which were inversely proportional to the number of compounds available within the training set and the number of targets within target expansion, respectively (Supplementary Figure S6).

**FIGURE 4:**
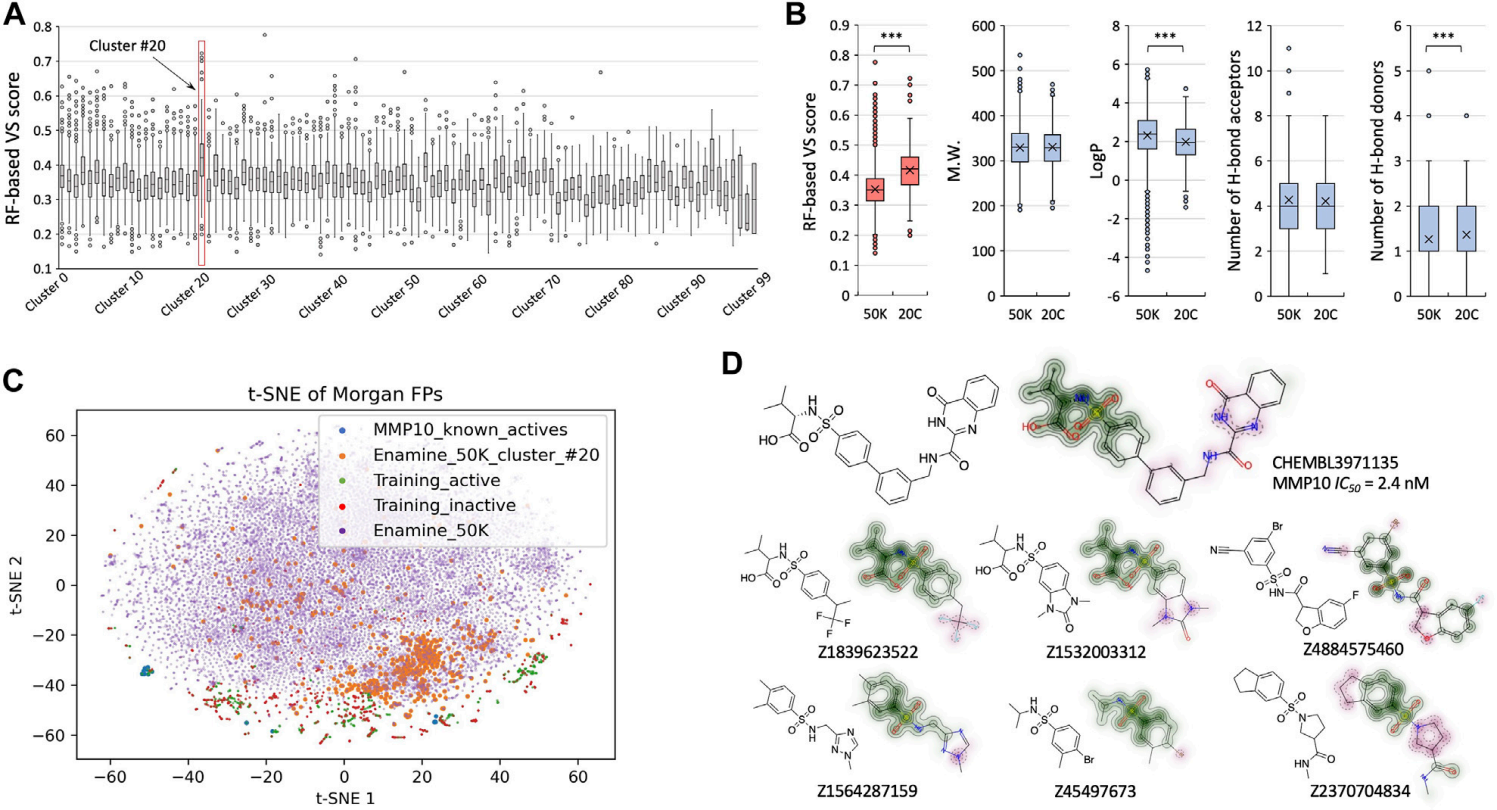
Structural insights revealed from the screening. **(A)**. Box and Whisker plots for RF-based VS scores across structurally clustered groups of Enamine 50K library. **(B)**. Comparison of VS scores and properties between cluster #20 and the remaining part of the Enamine 50K library. **(C)**. t-SNE analysis to visualize covered chemical spaces by known MMP10 actives (blue), cluster #20 (orange), active molecules in the training set (green), inactive molecules in the training set (red), and full Enamine 50K compounds (purple). **(D)**. One known MMP10 inhibitor and exemplified compounds in cluster #20. The overlapping benzenesulfonamide group is highlighted in green. Dissimilar moieties are colored pink.

To better understand the latent chemical space and structural insights that can be revealed from the process of virtual screening, we performed structural clustering and analysis for hits relating to stromelysin-2 (MMP10) from our retrospective studies (Figure 4). The Enamine 50K library was classified into one hundred structurally diversified clusters based on k-means clustering of encoded fingerprints, and we identified cluster #20 as having the greatest mean VS scores (Figure 4A). Interestingly, cluster #20 stood out as its upper extremes achieved comparable VS scores to known MMP10 active compounds (Figure 3B). Cluster #20 had a significant increase in mean RF-based VS score (0.42) compared to the remaining Enamine 50K library (0.35) (Figure 4B). From the perspective of physical–chemical properties, compounds in cluster #20 remained within the zone that follows the “rule of 5” (Lipinski et al., 1997) (Figure 4B). Subsequently, we performed t-SNE analysis to better visualize the chemical space coverage (Figure 4C). The compounds in the Enamine 50K library defined the overall boundary. Compounds in cluster #20 were largely concentrated as expected and partially overlapped with training molecules that were retrieved from the expanded target list. The known active MMP10 molecules were proximal to other training molecules but mostly independent from the compounds in cluster #20. Upon further investigation of specific chemical structures, it was found that compounds from cluster #20 that scored as highly active maintained a benzenesulfonamide group, which is a reported moiety of some known MMP10 inhibitors (Nara et al., 2016) (Figure 4D). This is an important finding, as known MMP10 active compounds were not included in the model training in retrospective studies. As seen in the retrieved molecules from the expanded target list, the TAME-VS platform detected chemical patterns in the training sets to construct a chemical understanding of the structure of the inhibitors. A Morgan fingerprint-based structural similarity search using the same known active MMP10 molecule as the query compound was conducted in parallel. Suggested molecules from our TAME-VS platform do not simply recur compounds with top Tanimoto coefficient (Tc) scores from the classical structural similarity search (Supplementary Figure S7A). TAME-VS can propose chemicals that align with the acquired structural patterns, even if they do not have high Tc similarity scores, which differs from the traditional approach of using structural similarity search (Supplementary Figure S7B, C). This observation further supported the claim that our TAME-VS platform can provide an alternative approach to tackle early-stage hit findings.

## 4 Discussion

The use of large-scale, high-throughput screening has been a major cornerstone of modern drug discovery efforts to identify chemical hits for novel protein targets and will be an important resource for the foreseeable future. Our novel TAME-VS platform enables users to survey chemical libraries rapidly and cost-effectively for *ab initio* drug discovery campaigns at a very early stage. This plug-and-play system provides a high degree of customizability and enables a broad range of users to explore desired chemical spaces to rapidly identify potential starting points for further chemical evaluation. Indeed, our retrospective validation across different protein types demonstrates a clear value in our platform with reliable predictive performance in the majority of cases.

We acknowledge that the use of the ChEMBL database limits the utility of the TAME-VS platform as low-homology or orphan proteins may not be represented within the database. However, this issue can be remedied by using the optional starting points, which allow users to flexibly supply their customized internal data, which may not be immediately available from public databases. For example, users can employ their own domain expertise to provide a more specified list of relevant targets for aggregating compound data in module 2, with *Compound Retrieval* serving as the optional *Starting Point #2* to begin the platform. Alternatively, if users have prepared their own compound lists from their internal experimental testing, module 3, *Vectorization*, can function as the optional *Starting Point #3* to leverage the remaining parts of the platform.

**FIGURE 5:**
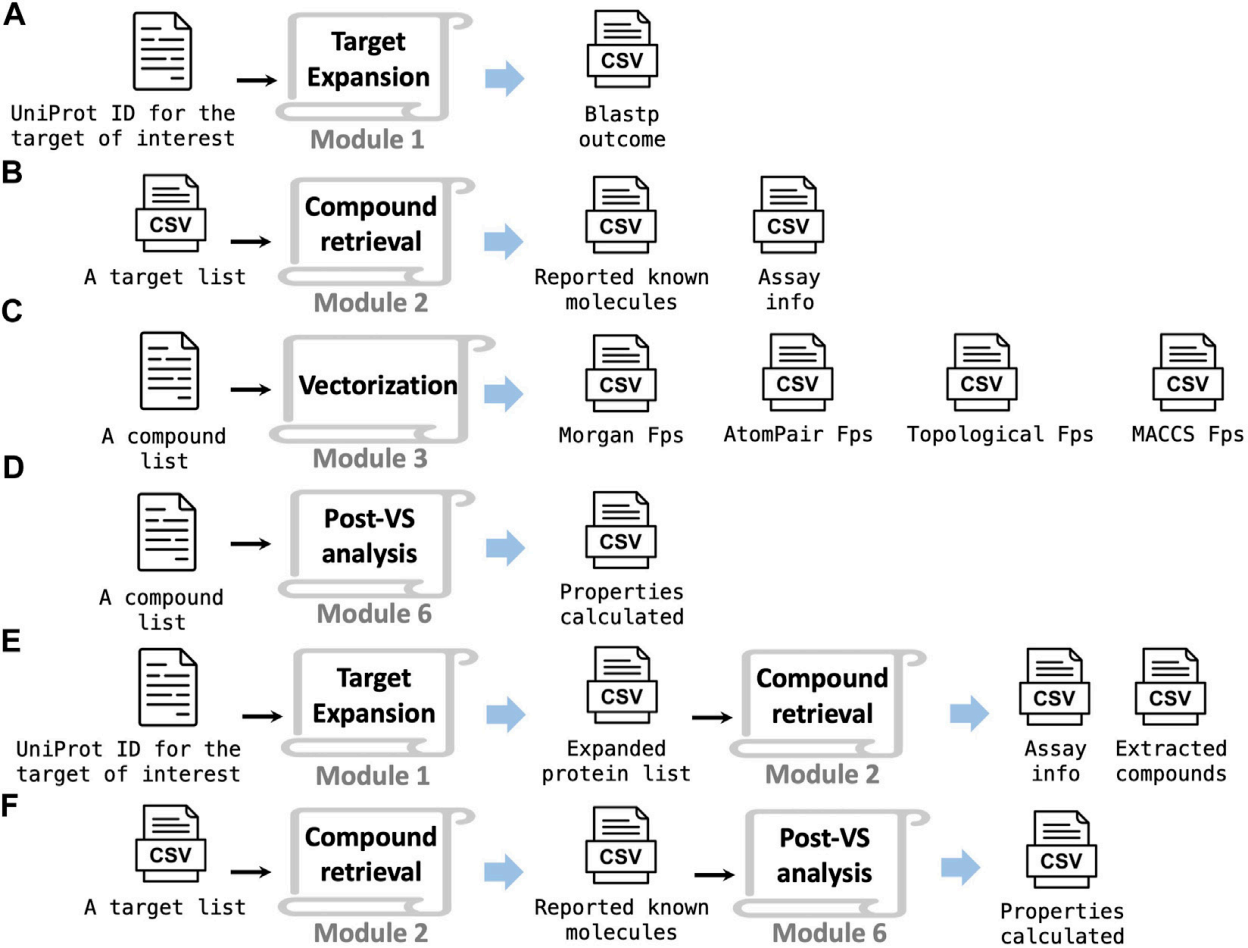
This platform has the flexibility for multi-purpose adaptation. Using an individual module **(A–D)** or a combination of modules **(E, F)** to realize various functions.

The TAME-VS platform serves as a relevant and flexible tool to efficiently perform virtual screening across a broad range of drug discovery stages. Furthermore, the platform can be deployed in a piecemeal fashion by running an individual module or a combination of multiple modules depending on user needs. Inside each module, there is a stand-alone Python script that can run independently with customized inputs and outputs. A Jupyter notebook for each module is also provided in case users prefer a more interactive experience. The following are a few examples. Module 1 provides an immediate solution to automated BLASTp search, which can improve sequence-focused bioinformatics studies (Figure 5A). Module 2 searches a large-scale compound collection for activity against given targets, which enables the creation of a focused chemical library for particular protein targets or target groups (Figure 5B). If a user requires a quick tool for fingerprints and key physical–chemical property calculation, module 3 (Figure 5C) and module 6 (Figure 5D) can be used for the task. For a just-initiated drug discovery project on a protein target, combining module 1 and module 2 can provide a bioinformatics overview of related, similar proteins and their corresponding interacting molecules (Figure 5E). Another example is combining module 2 and module 6 to calculate the chemical properties of molecules with activity against a given target (Figure 5F).

In addition to its flexibility in use, TAME-VS is also highly adaptable. There have been rapid advancements in ML applications and an ever-increasing expansion in AI methodologies for drug discovery (Vamathevan et al., 2019; Bian and Xie, 2021). Although we provide RF and MLP models to represent both classic ML algorithms and neural networks, respectively, additional add-on features can be appended in future updates to accommodate advancements in ML methods. Additionally, our platform can accommodate the use of novel molecular features and datatypes. Screening libraries can be customized, and post-screening analysis can integrate extra dimensions. The platform is highly customizable, easily integrated, and can be used to analyze data from multiple sources.

Our methodology provides a comprehensive, efficient, and flexible platform for virtual screening in target-driven drug discovery campaigns. It simplifies the process by streamlining the data processing, analysis, and visualization of results. This platform enables researchers to target novel proteins with limited starting information to rapidly evaluate and triage a large chemical space based on homology-expanded target lists and may help reduce the time and cost associated with launching a full drug discovery campaign. With its user-friendly programming environment, the TAME-VS platform can serve as an initial tool for early drug discovery and can increase the accessibility of these ML methods to a broad range of users.

## Data Availability

The open-source, freely available package of the TAME-VS platform is documented at https://github.com/bymgood/Target-driven-ML-enabled-VS.
