# Supplementary material for: Target-driven machine learning-enabled virtual screening (TAME-VS) platform for early-stage hit identification
Source: Front Mol Biosci. 2023 Mar 13;10:1163536. doi: 10.3389/fmolb.2023.1163536 (PMC10040869; doi:10.3389/fmolb.2023.1163536)
Supplement: Supplementary file 1 [file DataSheet1.DOCX]

Target-driven machine learning-enabled virtual screening (TAME-VS) platform for early-stage hit identification

Yuemin Bian^1, #, *^, Jason J. Kwon^2, 3, #^, Cong Liu^1^, Enrico Margiotta^1^, Mrinal Shekhar^1^, and Alexandra E. Gould^1^

^1^Center for the Development of Therapeutics, Broad Institute of MIT and Harvard, Cambridge, MA, USA.

^2^Cancer Program, Broad Institute of MIT and Harvard, Cambridge, MA, USA.

^3^Department of Medical Oncology, Dana-Farber Cancer Institute, Boston, MA, USA.

^#^These authors contributed equally to this work.

*** Correspondence:**Yuemin Bian, Ph.D.

Center for the Development of Therapeutics

Broad Institute of MIT and Harvard

415 Main Street

Cambridge, MA 02142, USA

Tel: +1 (412) 539-6258

Email: [ybian@broadinstitute.org](mailto:ybian@broadinstitute.org)

## SUPPLEMENTARY FIGURES


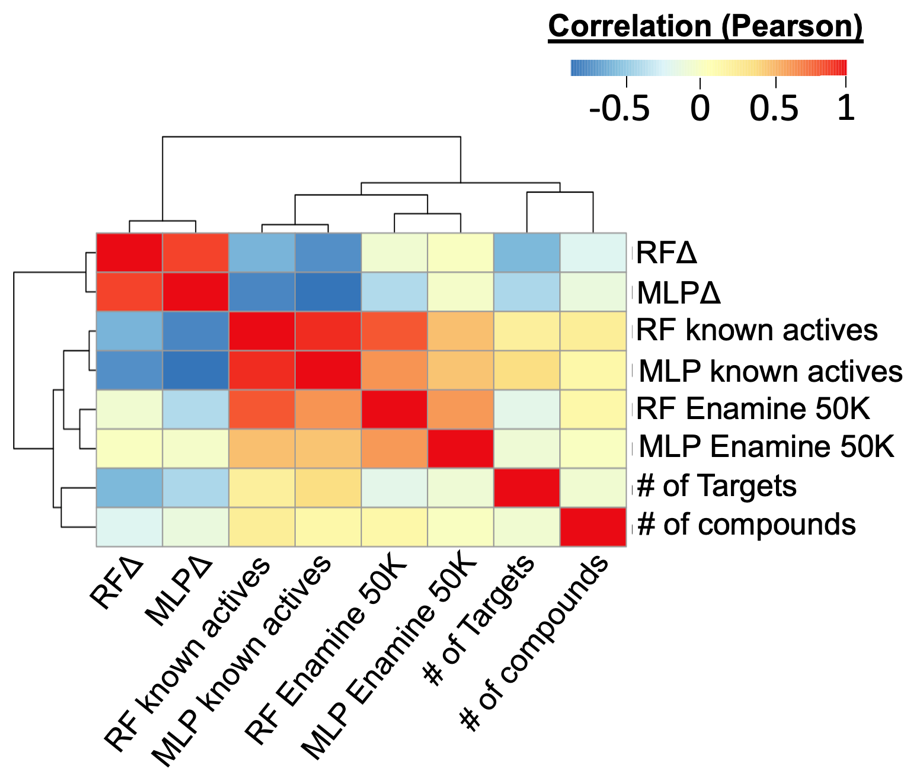


**Supplementary Figure 1. Correlation of model performance across ten target-based retrospective validations.** Correlation (Pearson) heatmap of attributes of the ten targets that were retrospectively evaluated: differential RF activity score between Known Actives and Enamine 50K (RFΔ); differential MLP activity score between Known Actives and Enamine 50K (MLPΔ); RF known active average score (RF known actives); MLP known active average score (MLP known actives); RF Enamine 50K average score (RF Enamine 50K); MLP Enamine 50K average score (MLP Enamine 50K); number of expanded targets in Module 1 (# of Targets); and number of compounds retrieved in Module 2 (# of compounds).

**
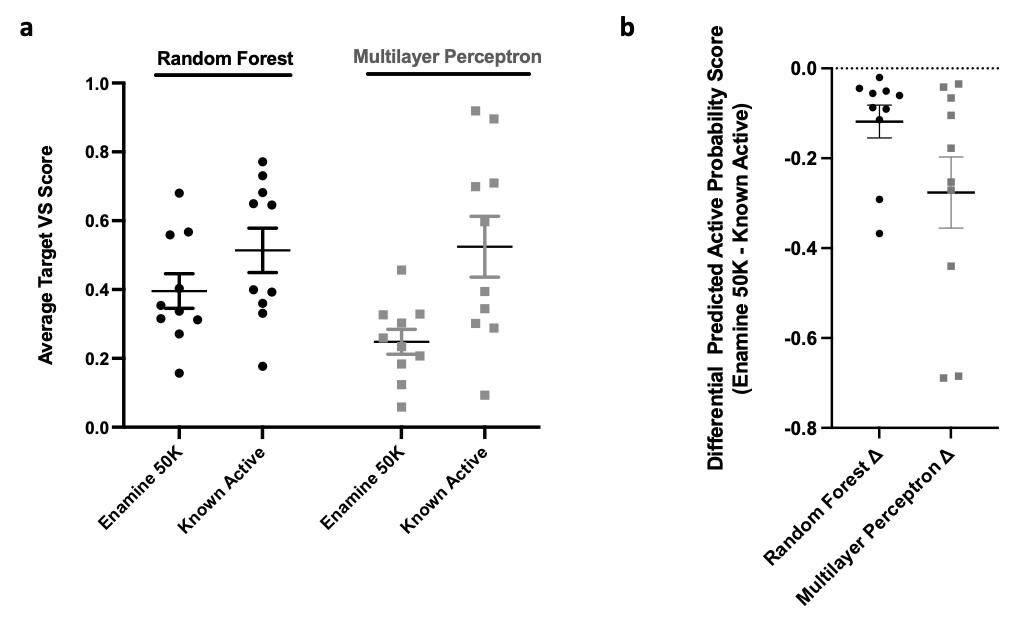
**

**Supplementary Figure 2. Average and differential VS Score analysis of 10 target-based retrospective validations** **a.** Scatter dot plot of the mean for each target VS score. Center bar represents mean, and error bars denote SEM. **b.** Scatter dot plot of the mean differential target VS score between Enamine 50K scores and known active compounds. Center bar represents mean, and error bars denote SEM.


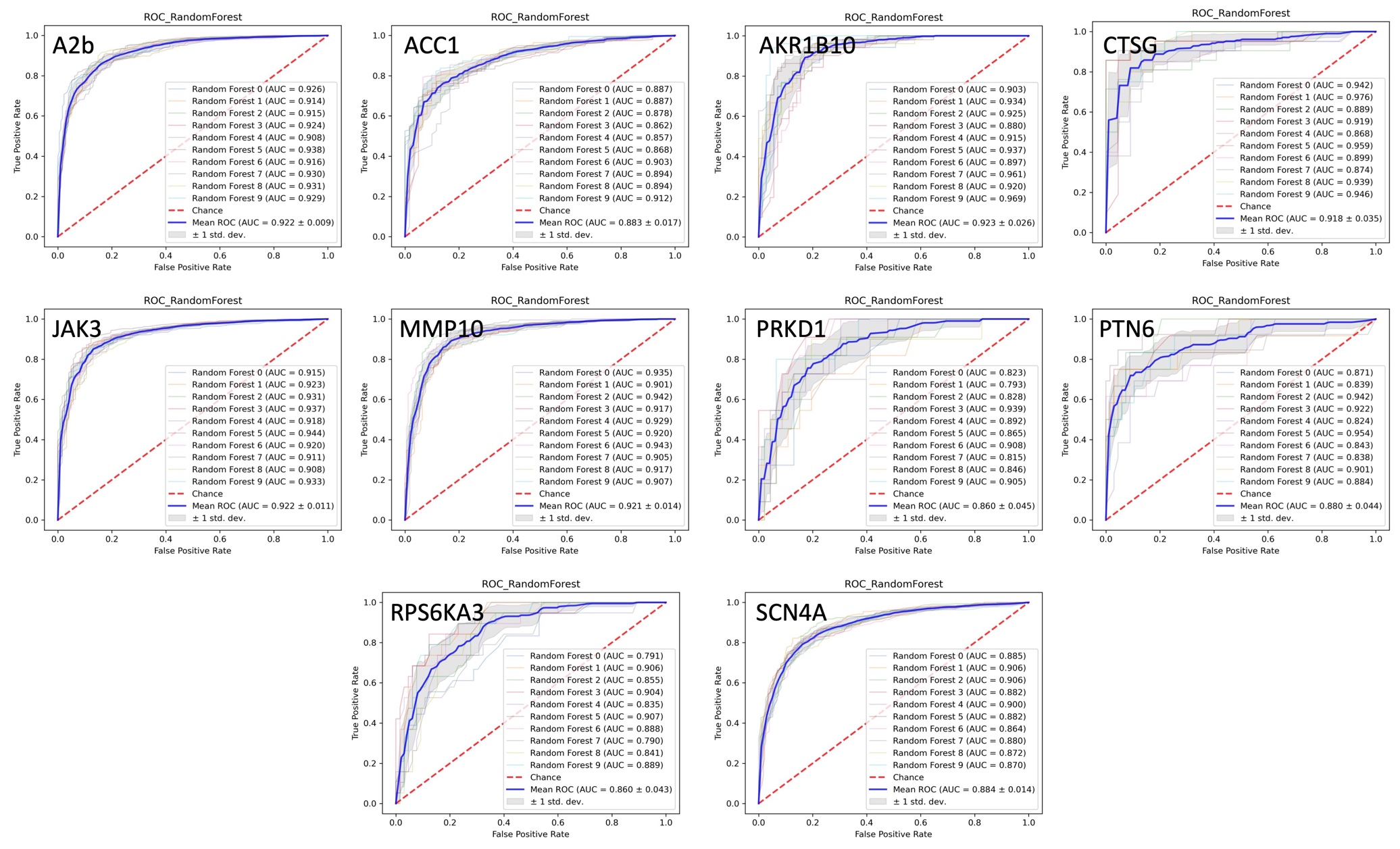


**Supplementary Figure 3. ROC curves for RF model training on ten protein targets with 10-time cross-validation**

**
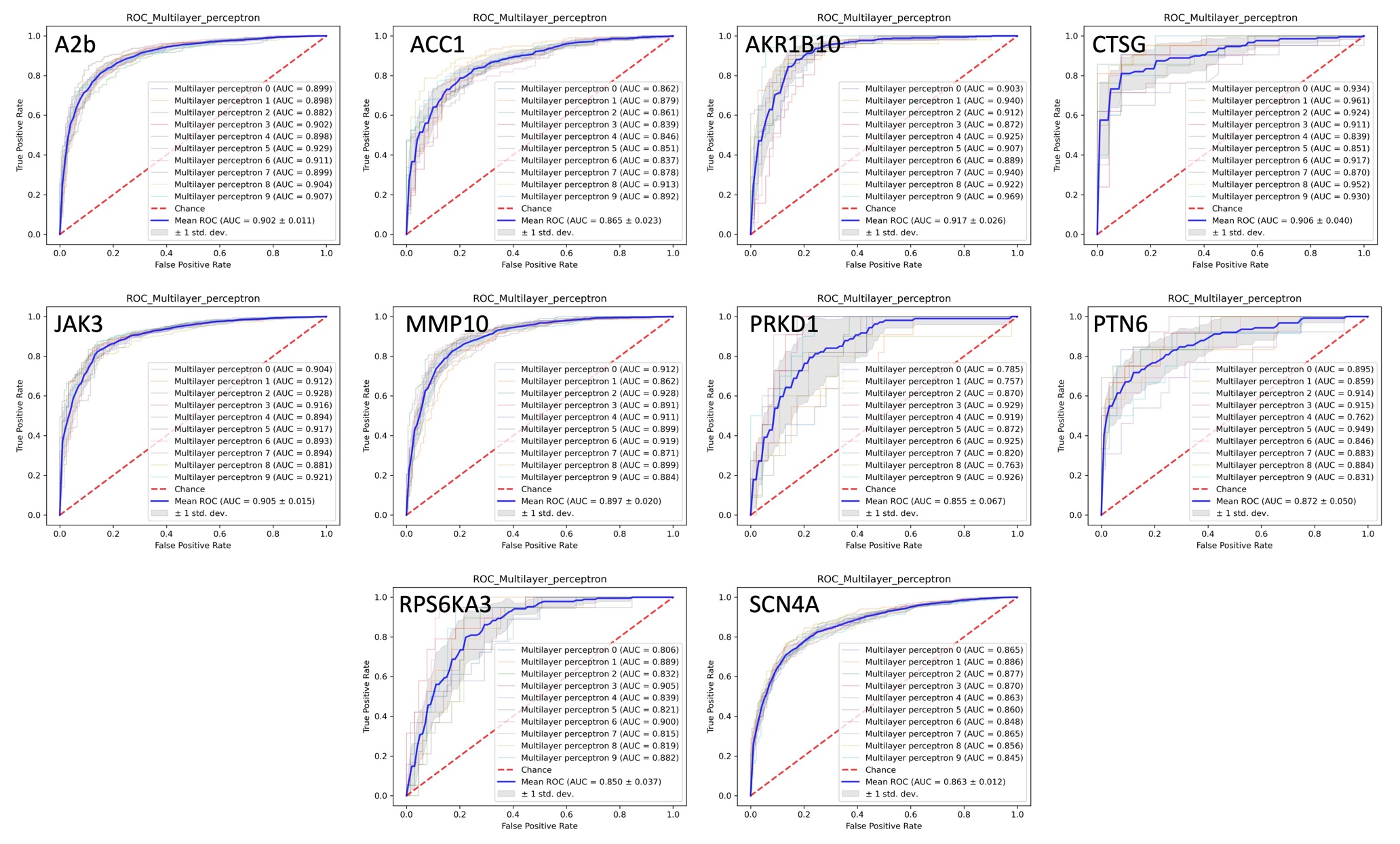
**

**Supplementary Figure 4. ROC curves for MLP model training on ten protein targets with 10-time cross-validation.**


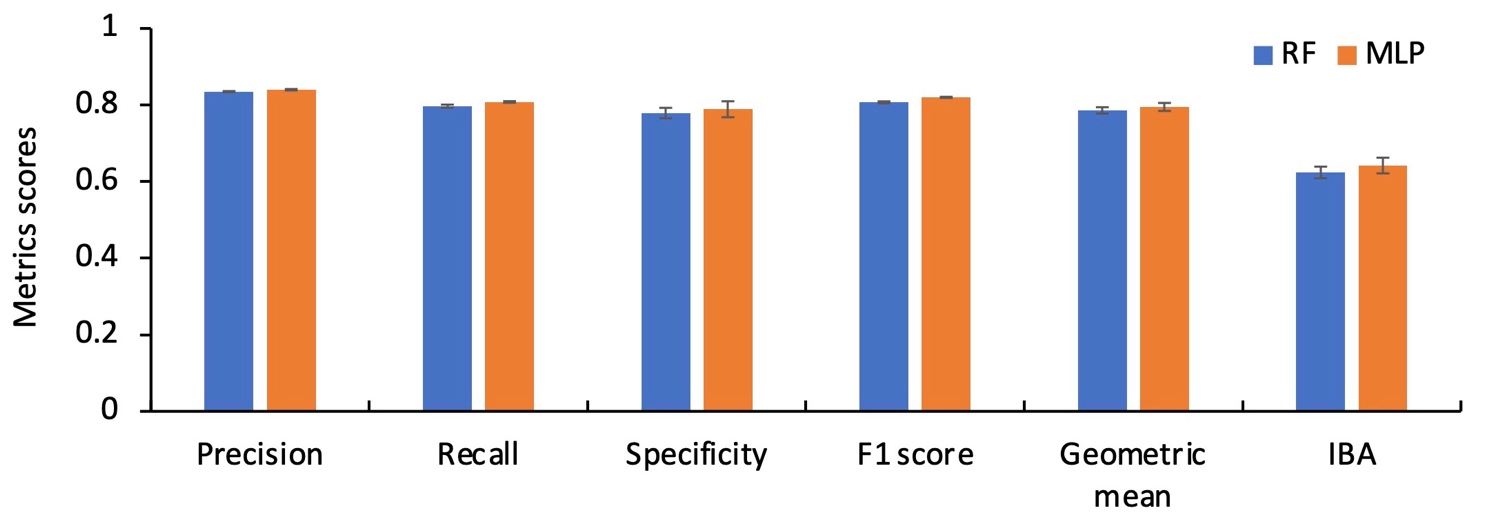


**Supplementary Figure 5. Bar chart of averaged metrics from ten RF and MLP models.** The error bar represents variance. IBA stands for index of balanced accuracy.


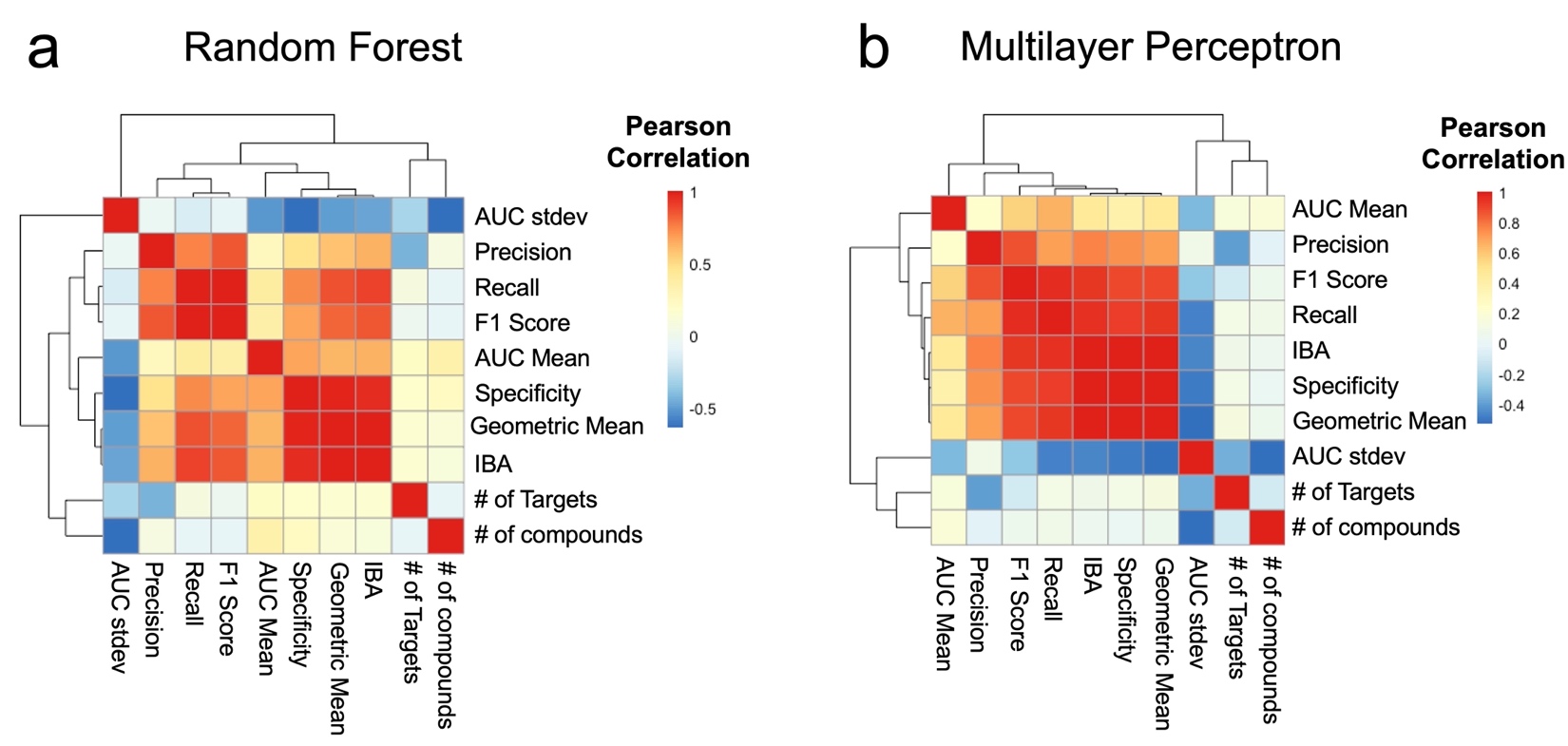


**Supplementary Figure 6. Correlation of model metrics across ten target-based retrospective validations.** Correlation (Pearson) heatmap of attributes of the ten targets that were retrospectively evaluated: averaged precision, averaged recall, averaged specificity, averaged F1 score, averaged geometric mean, and averaged IBA for both (**A**) Random Forest and (**B**) Multilayer Perceptron.


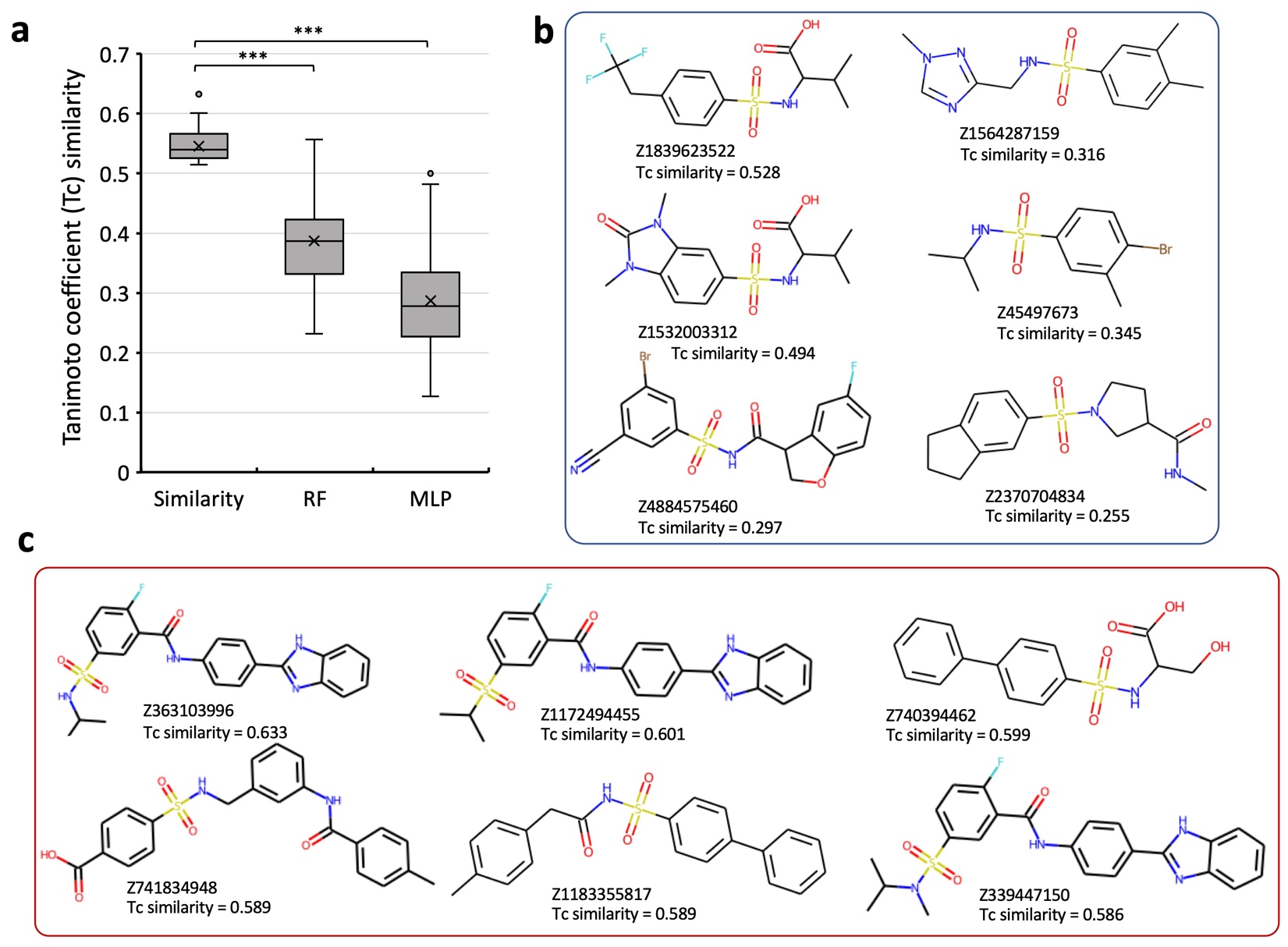


**Supplementary Figure 7. A comparison of top molecules suggested from the TAME-VS and compound structural similarity search.** **a**. The box and whisker plot to compare the Tanimoto coefficient (Tc) similarity towards the known MMP10 inhibitor CHEMBL3971135 of top 100 suggested compounds from ligand-based similarity search and TAME-VS models (RF and MLP). **b**. Exemplified compounds in cluster #20 with Tc similarities calculated. **c**. Exemplified compounds with top Tc similarities revealed from the ligand-based similarity search.

## SUPPLEMENTARY TABLES

**Supplementary Table 1. Calculated metrics of the RF classifier across ten targets**

| **Target** | **AUC (mean)** | **AUC (stdev)** | **Precision** | **Recall** | **Specificity** | **F1 score** | **Geometric mean** | **Index of balanced accuracy** |
| --- | --- | --- | --- | --- | --- | --- | --- | --- |
| A2b | 0.92 | 0.01 | 0.84 | 0.82 | 0.84 | 0.82 | 0.83 | 0.68 |
| ACC1 | 0.88 | 0.02 | 0.83 | 0.77 | 0.83 | 0.78 | 0.8 | 0.63 |
| AKR1B10 | 0.92 | 0.03 | 0.82 | 0.82 | 0.82 | 0.82 | 0.82 | 0.67 |
| CTSG | 0.92 | 0.04 | 0.83 | 0.82 | 0.83 | 0.82 | 0.83 | 0.68 |
| JAK3 | 0.92 | 0.01 | 0.86 | 0.76 | 0.84 | 0.79 | 0.8 | 0.64 |
| MMP10 | 0.92 | 0.01 | 0.85 | 0.84 | 0.85 | 0.84 | 0.85 | 0.72 |
| PRKD1 | 0.86 | 0.05 | 0.79 | 0.66 | 0.46 | 0.71 | 0.54 | 0.30 |
| PTN6 | 0.88 | 0.04 | 0.92 | 0.91 | 0.80 | 0.91 | 0.85 | 0.73 |
| RPS6KA3 | 0.86 | 0.04 | 0.82 | 0.79 | 0.74 | 0.8 | 0.76 | 0.58 |
| SCN4A | 0.88 | 0.01 | 0.79 | 0.78 | 0.78 | 0.78 | 0.78 | 0.61 |

**Supplementary Table 2. Calculated metrics of the MLP classifier across ten targets**

| **Target** | **AUC (mean)** | **AUC (stdev)** | **Precision** | **Recall** | **Specificity** | **F1 score** | **Geometric mean** | **Index of balanced accuracy** |
| --- | --- | --- | --- | --- | --- | --- | --- | --- |
| A2b | 0.90 | 0.01 | 0.84 | 0.82 | 0.83 | 0.83 | 0.82 | 0.68 |
| ACC1 | 0.87 | 0.02 | 0.82 | 0.79 | 0.77 | 0.8 | 0.78 | 0.61 |
| AKR1B10 | 0.92 | 0.03 | 0.82 | 0.82 | 0.82 | 0.82 | 0.82 | 0.67 |
| CTSG | 0.91 | 0.04 | 0.86 | 0.85 | 0.87 | 0.85 | 0.86 | 0.74 |
| JAK3 | 0.91 | 0.02 | 0.86 | 0.83 | 0.8 | 0.84 | 0.82 | 0.67 |
| MMP10 | 0.90 | 0.02 | 0.85 | 0.85 | 0.85 | 0.85 | 0.85 | 0.72 |
| PRKD1 | 0.86 | 0.07 | 0.79 | 0.71 | 0.42 | 0.75 | 0.53 | 0.29 |
| PTN6 | 0.87 | 0.05 | 0.94 | 0.85 | 0.98 | 0.88 | 0.91 | 0.83 |
| RPS6KA3 | 0.85 | 0.04 | 0.82 | 0.76 | 0.75 | 0.78 | 0.76 | 0.57 |
| SCN4A | 0.86 | 0.01 | 0.80 | 0.80 | 0.80 | 0.80 | 0.80 | 0.64 |

## SUPPLEMENTARY INFO

Metrics calculated for evaluating RF and MLP models:

Precision (Pre): Precision is the number of true positive predictions divided by the sum of true positive and false positive predictions. It measures the accuracy of positive predictions.

Recall (Rec): Recall is the number of true positive predictions divided by the sum of true positive and false negative predictions. It measures the completeness of positive predictions.

Specificity (Spe): Specificity is the number of true negative predictions divided by the sum of true negative and false positive predictions. It measures the accuracy of negative predictions.

F1 Score (F1): F1 Score is the harmonic mean of precision and recall. It is a balance between precision and recall and is a commonly used metric in imbalanced datasets.

Geometric Mean (Geo): Geometric Mean is the geometric average of precision and recall. It is a balance between precision and recall and is particularly useful in imbalanced datasets, as it is sensitive to both false positive and false negative errors.

Index balanced accuracy (IBA): IBA is a measure of the overall performance of a binary classifier that takes into account both precision and recall, as well as the imbalance in the distribution of classes.
